# Role of PON in Anoxia-Reoxygenation Injury: A Drosophila Melanogaster Transgenic Model

**DOI:** 10.1371/journal.pone.0084434

**Published:** 2014-01-06

**Authors:** Juan Carlos Caraballo, Jennifer Borcherding, Michael Rector, Emma Hornick, David Stoltz, Joseph Zabner, Alejandro P. Comellas

**Affiliations:** Department of Internal Medicine, University of Iowa, Iowa City, Iowa, United States of America; CINVESTAV-IPN, Mexico

## Abstract

**Background:**

Paraoxonase 1 (PON1) is a protein found associated with high density lipoprotein (HDL), thought to prevent oxidative modification of low-density lipoprotein (LDL). This enzyme has been implicated in lowering the risk of cardiovascular disease. Anoxia-reoxygenation and oxidative stress are important elements in cardiovascular and cerebrovascular disease. However, the role of PON1 in anoxia-reoxygenation or anoxic injury is unclear. We hypothesize that PON1 prevents anoxia-reoxygenation injury. We set out to determine whether PON1 expression in *Drosophila melanogaster* protects against anoxia-reoxygenation (A-R) induced injury.

**Methods:**

Wild type (WT) and transgenic PON1 flies were exposed to anoxia (100% Nitrogen) for different time intervals (from 1 to 24 hours). After the anoxic period, flies were placed in room air for reoxygenation. Activity and survival of flies was then recorded.

**Results:**

Within 5 minutes of anoxia, all flies fell into a stupor state. After reoxygenation, survivor flies resumed activity with some delay. Interestingly, transgenic flies recovered from stupor later than WT. PON1 transgenic flies had a significant survival advantage after A-R stress compared with WT. The protection conferred by PON1 expression was present regardless of the age or dietary restriction. Furthermore, PON1 expression exclusively in CNS conferred protection.

**Conclusion:**

Our results support the hypothesis that PON1 has a protective role in anoxia-reoxygenation injury, and its expression in the CNS is sufficient and necessary to provide a 100% survival protection.

## Introduction

Paraoxonases (PON) are a family of enzymes whose enzymatic activity is not well understood. There are three known members of the PON family in humans, named PON1, PON2 and PON3. PON1 is a 45 kDa glycoprotein expressed in different tissues including kidney, colon, and the liver and mainly found associated with HDL in the bloodstream which in turn can deliver the protein to various tissues. Epidemiological data shows an association between PON1 activity, systemic oxidative stress and cardiovascular risk in humans [1], [2]. Furthermore, PON1 is thought to be responsible for the antioxidant effect of HDL as it inhibits lipid peroxidation and mediates cholesterol efflux from atherosclerotic plaque [3]. PON1 can be localized in endothelial cells and it has been shown to be necessary and sufficient for preventing LDL oxidation [4], [5], [6]

Reperfusion of hypoxic/anoxic tissues induces a massive production of reactive oxygen species (ROS) causing important oxidative damage [7]. Oxidative damage is central in the pathophysiology of anoxia-reoxygenation (AR) injury, such as in myocardial infarction, ischemic stroke and organ transplant. For example, Ferretti and colleagues reported that paraoxonase activity was lower in stroke patients compared to controls, and this correlated to a higher level of lipid peroxidation [8]. In addition, reduced PON1 activity is an important risk factor for atherosclerosis and serum PON1 activity also predicts arterial stiffness in renal transplant recipients [9].

Although most epidemiological studies suggest that PON1 protection against cardiovascular disease is due to its antioxidant effects, little is known about the role of this protein in anoxia-reoxygenation injury and other oxidative-mediated pathologies.

Determining the role of PON1 in different injury models is challenging, as in the mammalian systems there are three PONs, therefore, to avoid the potential of compensatory mechanisms in a PON1 knockout, a triple knockout (PON 1,2,&3) is required. One strategy that other investigators have developed to overcome the above challenge is testing the role of PON in an invertebrate model, specifically, *Drosophila melanogaster*, as flies do not contain the PON gene or any homologs [10], [11]. This approach eliminates the variable of redundancy.

We decided to test our hypothesis in model of anoxia-reoxygenation (AR) in *Drosophila melanogaster*, as a model of AR injury as previously reported by Vigne and colleagues[12].

We hypothesized that PON1 expression will protect flies from AR injury. To test this hypothesis we conducted a series of experiments where transgenic flies expressing tubulin (WT) and flies expressing PON1 were exposed to AR injury. We demonstrated that PON1 expression confers a survival advantage against AR injury, and specifically PON1 expression in the CNS protects against AR induced mortality.

## Methods

### Experimental animals

All flies were maintained and cultured on standard yeast-agar-sucrose-cornmeal medium. Flies were bred and tested at 25°C. The binary GAL4-UAS system and *tub* promoter was used for the ubiquitous transgenic expression of PON1 (3). The neuron specific Elav driver and gut driver were used instead of tub promoter to achieve expression of PON1 exclusively in the CNS and gut, respectively.

Drosophila stocks were reared on standard cornmeal-agar-molasses medium at room temperature (21–25°C). The tubulin-Gal4 transgenic line (y1 w*; P{tubP-GAL4}LL7/TM3, Sb1) was obtained from the Bloomington stock center (Bloomington, Indiana).

### Anoxia exposure

**Figure 1:**
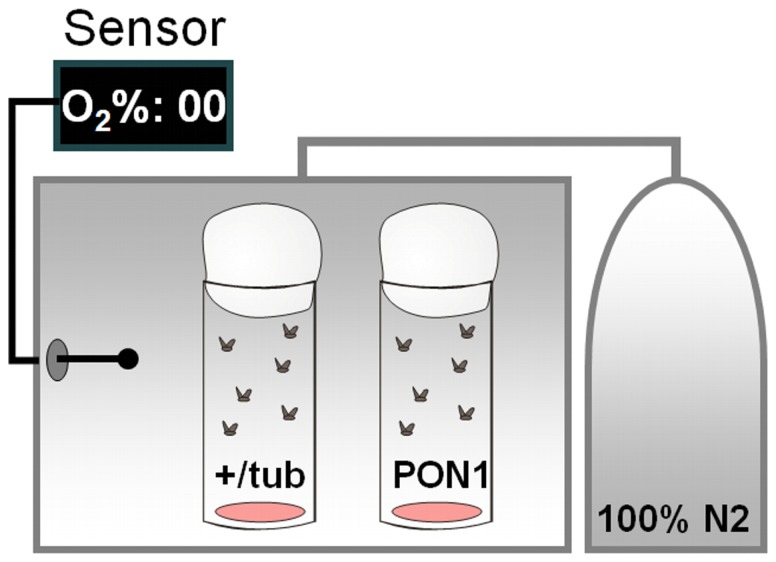
Schematic representation of anoxic chamber.

+/tub and PON1 transgenic flies were placed in vials (20 flies per vial) inside a plastic chamber (see Figure 1). 100% Nitrogen was injected in the chamber until oxygen levels dropped below 1%. Oxygen level was monitored with an oxymeter (TED 200-T Portable oxygen monitor, Teledyne electronic devices (Thousand Oaks, California). Within 1 min of anoxia, flies stop moving and fall into anoxia-induced stupor (paralysis). After different time periods, flies were placed at room air for reoxygenation, allowing them to recover from anoxia-induced stupor. Survival was registered 48 hours later.

In a subset of flaks exposed to 1h of anoxia, flasks were examined every 5 min after exposure to rom air and number of active flies were recorded. All flies that remained motionless at the bottom of the flask were considered inactive, and all flies that were moving or at the wall of the flask were considered active.

### ROS measurement method

Guts from flies were dissected and stained using Dihydroethidium (DHE), a superoxide indicator. Guts were then observed under fluorescence microscope.

The entire gastrointestinal tract was removed intact from females for staining and further processing by grasping below the head and at the tip of the abdomen with fine forceps and gently pulling.

Female adult fly guts (3–5 days old, from same parents and bottle) were dissected as previously described under 1× Schneider's Drosophila Media (Gibco, Invitrogen, Carlsbad, CA). The guts were then stained with DHE using a previously described method [13].

To assess superoxide (O_2_^−^) levels in whole flies, a lucigenin based ROS assay was performed on fly lysate as previously described[14], [15]. Fly lysates were prepared using 3-day old male whole flies. For each sample 3 flies were homogenized with a pestle in mitochondria buffer (1mM Tris pH 7.5, 20 µM EDTA, Aprotinin 2 ng/mL, Leupeptin 2 ng/mL, Pepstatin 2ng/mL). After sonication, lysates were centrifuged and supernatant collected. A Bradford assay was completed on all samples prior to conducting the experiment. 50 µg of protein were diluted in 1× PBS to a final volume of 1ml. Lucigenin (5 µM) and NADPH (100 µM) (Sigma-Aldrich, St. Louis, MO) were added to each sample, and luminescence was recorded every 30 seconds (s) for 10 min. Initial rate was defined as the linear slope of the data points from 30 s to 150 s.

### Generation of Transgenic Fly Lines

The y w^1118^ strain was used for transgenic injections. P element-mediated transformation and subsequent fly crosses were performed following standard techniques [16]. To generate UAS-PON1 transgenics the cDNA sequence for PON1 was cloned into the pUAST vector.

The binary GAL4-UAS system and tubulin (tub) promoter were used for the ubiquitous transgenic expression of PON1. Human PON1 cDNA was cloned into the pUAST plasmid and subsequently injected into *Drosophila melanogaster* embryos (y w^1118^) using standard techniques (Rainbow Transgenic Flies, Inc. Camarillo, CA). y w^1118^; ; tub-GAL4/TM3,Sb virgin females were crossed to y w^1118^; ; UAS-PON1 males to obtain the experimental lines y w^1118^; ; UAS-PON1/tub-GAL4, which is referred to as UAS-PON1/tub-GAL4. Control flies (w^1118^; tub-GAL4/+) were obtained by crossing w^1118^ males with y w^1118^; tub-GAL4/TM3, Sb virgin females and are denoted as tub/+. F1 progeny were tested for expression of PON1 by performing western blots using an antibody to PON1 (Abcam, Cambridge, MA). The neuron specific Elav driver and gut driver were used instead of tubulin driver to achieve expression of PON1 exclusively in the CNS and gut, respectively.

### Statistical analysis

All experiments were performed at least 3 times separately with 20 flies per condition per experiment. Statistical analyses were performed in Graphpad Prism 5. Unpaired t-test, paired t-test and Mann-Whitney U test were performed as indicated. A p-value<0.05 was considered statistically significant [17].

## Results

### PON1 expression increases survival after AR

Vinge and colleagues have reported that mortality increases in *Drosophila* after AR exposure [12]. Specifically, mortality increased acutely during the first two days and remained the same for the rest of their life span. We decided to test whether PON1 expression in *Drosophila melanogaster* protected against AR induced mortality. Wild type +/tub flies (WT) and transgenic flies expressing Paraoxonase 1 (PON1) were exposed to different periods of anoxia (1, 2, & 4 hours). After anoxic period, flies were placed at room air for reoxygenation. Survival was recorded 48h after AR stress. Oxygen levels of <1% were achieved after 5 min of injecting 100% Nitrogen into the chamber. Within one minute of achieving this level of anoxia, flies stop flying and lay at the bottom of the vial, motionless. They remained stuporous throughout the anoxic time. After reoxygenation, flies became active from the anoxia-induced stupor and start flying.

**Figure 2:**
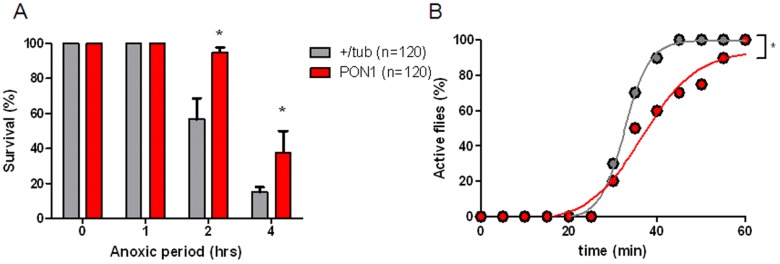
Survival and recovery from stupor after anoxia-reoxygenation. +/tub and PON1 flies were exposed to anoxia for different time periods 1–4 hours. A) survival registered 24 hours after reoxygenation. B) percentage of flies that recover from stupor over time. *p<0.05 when compared with +/tub.

As shown in Figure 2A, survival decreased as the anoxic period increased. PON1 transgenic flies showed a survival advantage of around 20% compared to WT flies. 100% of flies exposed to one and two hours of anoxia survived, however, PON1 flies had a longer stupor recovery time compared to WT (Figure 2B).

### PON1 R and Q protect against AR induced mortality via a decrease in baseline ROS levels

**Figure 3:**
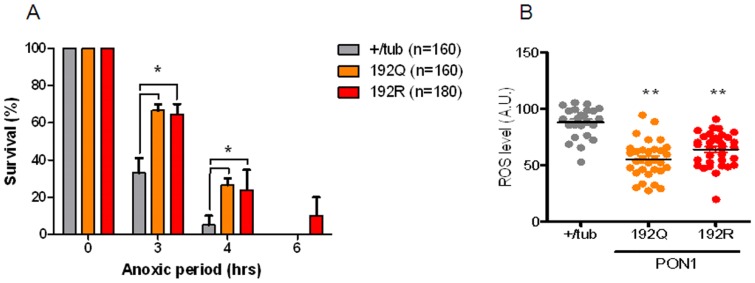
Survival and ROS of transgenic flies expression PON1 variants. A) +/tub, 192Q and 192R transgenic flies were exposed to different anoxia periods, survival was registered 24 hrs after reoxygenation. B) ROS level in flies measured with dihydroethdium staining. *p<0.05, ** p<0.01 when compared with +/tub.

One common polymorphism is at position 192, were a glutamine (192Q) is replaced by an arginine (192R). These alloenzymes have differences in their paraoxonase and arylesterase activity [18]. Epidemiological data shows an association between different PON1 polymorphisms and cardiovascular risk [1]. Bhattacharyya and colleagues reported that subjects with QQ192 polymorphism have significantly increased levels of oxidative stress and higher risk of all-cause mortality compared with RQ192 and RR192. However, other studies have failed to demonstrate this association [19]. We hypothesized that PON1 192R will provide higher protection from AR injury compared with 192Q. To test this hypothesis, we exposed WT, 192Q and 192R transgenic flies to different periods of anoxia. As shown in the Figure 3A, both Q and R, confer similar survival advantage after three and four hours of anoxia. Only R provided a survival advantage of 10% after 6 hours of AR (see Figure 3A).

Since the mechanisms of AR injury are mediated by ROS, guts from flies of each variant were stained using Dihydroethidium, a superoxide indicator. Figure 3B shows that the level of superoxide in both, 192Q and 192R transgenic flies are lower compared with WT transgenic flies.

### Age and dietary restriction does not affect survival advantage conferred by PON1 expression

**Figure 4:**
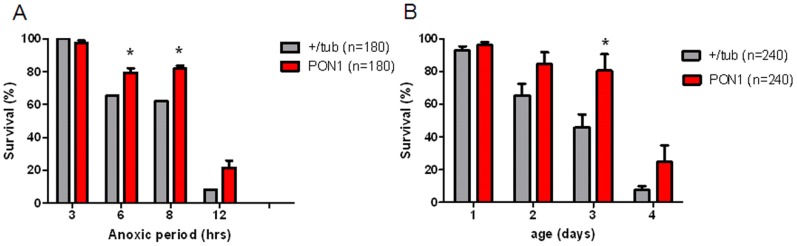
Survival of transgenic flies after starvation period. A) +/tub and PON transgenic flies were starved for 18 hrs and then exposed to different periods of anoxia, survival was registered 24 hrs after reoxygenation. B) +/tub and PON transgenic flies of different ages were exposed to 3 hrs of anoxia and survival was registered 24 hrs after reoxygenation. *p<0.05 when compared with +/tub.

Transgenic flies have a slight difference in food intake of about 10% (data not shown), and since dietary restriction increases survival after AR [12], we exposed PON1 and WT flies to anoxia after 18 hours of starvation, having access to water only, in order to investigate the role of PON1 expression regardless of food intake. As shown in Figure 4A, starvation confers a significant increase in survival in WT and PON1 flies, and longer periods of anoxia were necessary to increase mortality. Nevertheless, at higher anoxic periods (6, 8, & 12 hours), PON1 flies continue to have survival advantage.

We also investigated whether aging will influence AR induced mortality. Flies were paired to ages ranging from one to four days and exposed to three hours of anoxia. 48h survival was registered. As shown in Figure 4B, survival to AR decreases with age in both WT and PON1, however, PON1 transgenic flies continued to have a survival advantage compared with WT despite controlling for age effect.

### PON1 expression in the Central Nervous System (CNS) confers survival advantage from AR

**Figure 5:**
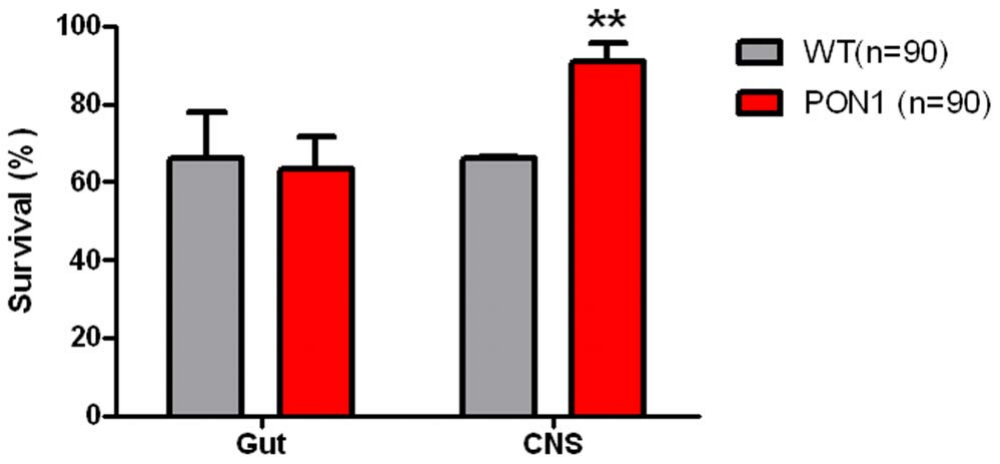
Survival of transgenic flies expressing PON with organ specific promoters. +/tub and PON transgenic flies with organ specific promoters were exposed to 3 hrs of anoxia and survival was recorded 24 hrs later. *p<0.05, ** p<0.01 when compared with +/tub.

In order to determine whether regional expression of PON1 would confer survival advantage against AR, we developed two strains of flies with organ specific promoters that expressed PON1 protein only in the central nervous system (CNS) or in the Gut. In a similar manner of previous experiments, flies were exposed to three hours of anoxia and survival was registered 48 hours later. PON1 CNS transgenic flies have higher survival than +/tub CNS flies, similar to transgenic flies with a universal expression of PON1, while flies expressing PON1 in the gut survived similarly to WT flies (Figure 5).

## Discussion

Oxidative stress is an essential element of ischemia reperfusion injury, where perfusion of anoxic tissues produces an increase of ROS production beyond the antioxidant capability of the organism causing oxidative damage of the cell membrane by lipid peroxidation. This is the hallmark of multiple pathological processes including myocardial infraction, stroke, and transplant. During the last decade, paraoxonase enzymes have been shown to be an important element in the physiopathology of oxidative stress. It is well known that PON1 prevents the oxidation of LDL, and it is necessary for the antioxidant properties of HDL on LDL [3], [4], [20]. Furthermore, Rozenberg and colleagues showed that lack of PON1 increases oxidative stress in macrophages [21]. It has been shown that PON activity correlates with cardiovascular risk and atherosclerotic disease, which results from oxidation of LDL and accumulation in artery walls [22]. Although native enzymatic activity of PON1 is unclear, there is growing data supporting the role of PON enzymes as antioxidants. However, there is a paucity of data regarding the role of PON enzymes as antioxidants following anoxia-reoxygenation injury. The results of our study suggest that PON1 plays a protective role in anoxia-reoxygenation.

PON1 activity has been implicated in having a protective effect in ischemia colitis [23] and more recently, it has been shown that protective effects of dexmedetomidine on liver ischemia reperfusion was associated with an increase in PON1 activity [24]. Okur and colleagues recently reported that patients with obstructive sleep apnea, non-apneic and nocturnal desaturated COPD had increased levels of lipid peroxidation and decreased PON activity despite the differences in nocturnal hypoxia pattern.

Anoxia-reoxygenation, as well as ischemia reperfusion injury, is mainly mediated by the overproduction of ROS. Our results show that transgenic expression of PON1 in *Drosophila melanogaster* confers a survival advantage against AR injury. Our results also show that flies expressing PON have lower levels of superoxide at baseline, suggesting that PON1 has antioxidant effects, protecting flies from oxidative stress after anoxia-reoxygenation. PON activity not only prevents oxidative stress that promotes atherosclerotic plaque formation but also may be relevant in more rapid burst of oxidative stress as in AR injury. More recently, it has been reported that PON1 forms a complex with HDL and myeloperoxidase, an important oxidative enzyme, partially inhibiting myeloperoxidase activity [25].

Transgenic flies had a delayed recovery from AR-induced stupor. This effect can potentially explain some of the protection conferred by PON1 expression, as flies are extremely resistant to hypoxia and anoxia, and they respond to low oxygen and anoxia by decreasing their activity, and oxygen utilization [26]. This delay in activity can buffer the ROS production in PON1 transgenic flies, thus improving their survival.

Also, our results suggest that protection is due to PON1 expression in the brain; as expression of PON1 in flies was sufficient and necessary for protection against AR injury in the same proportion that universal expression of PON1. In humans, PON1 mRNA has been found mainly in kidney, colon and liver [27], but protein is more widely distributed [28]. For example, PON1 can be transferred from HDL to the cell membrane of different tissues, including the brain [20].

Epidemiological data shows a clear association of ischemic stroke incidence, survival and PON activity, where subjects with 192R have a higher risk for ischemic stroke than 192Q [29], [30]. However, the data regarding the role of this polymorphism and cardiovascular risk is conflicting. Some studies report significant risk of CAD and higher level of oxidative stress with variant 192R [31], [32], while others show no association [33], [34]. Also, PON polymorphism has been associated with difference in response to clopidogrel, where loss of function alleles increased risk of having major cardiovascular event in patients on clopidogrel treatment [35]. In our study, flies expressing 192R and 192Q have similar ROS levels and both have similar survival protection against AR injury.

PON1 is only one of three enzymes in the family of PON in humans. PON are promiscuous enzymes and its native enzymatic function is not clear. PON1 is the only one with paraoxonase activity, while all members of the family have different degree of lactonases and acetylesterase activity, whose relevance for antioxidant effect remains to be determined. While PON1 is expressed mainly in the liver and found associated to HDL, PON2 is ubiquitously expressed and its role in oxidative damage by anoxia reoxygenation is potentially more relevant and it still remains to be tested. Giordano G and colleagues reported that PON2 is expressed in the brain and protects CNS cells against oxidative stress and confers gender-dependent susceptibility to such stress [36], [37]. This suggests that both enzymes share similar enzymatic activity that protects against oxidative stress in the CNS. Further experiments, comparing PON1 and other members of the PON family with different enzymatic activity may help elucidate the role of lactonase and acetylesterase activity. In addition, it is necessary to explore the role of PON in AR injury in mammalian models, to help determine the role of these enzymes in humans.
